# RNF25 promotes gefitinib resistance in EGFR-mutant NSCLC cells by inducing NF-κB-mediated ERK reactivation

**DOI:** 10.1038/s41419-018-0651-5

**Published:** 2018-05-22

**Authors:** Jung Hee Cho, Yeon-Mi You, Y I Yeom, Dong Chul Lee, Bo-Kyung Kim, Misun Won, Byoung Chul Cho, Minho Kang, Seulki Park, Suk-Jin Yang, Jang Seong Kim, Jung-Ae Kim, Kyung Chan Park

**Affiliations:** 1 Personal Genomic Medicine Research Center, Korea Research Institute of Bioscience and Biotechnology, Daejeon, 34141 South Korea; 2 Biotherapeutics Translational Research Center, Korea Research Institute of Bioscience and Biotechnology, Daejeon, 34141 South Korea; 3 Department of Functional Genomics, University of Science and Technology, Daejeon, 34113 South Korea; 4 Division of Medical Oncology, Yonsei Cancer Center, Yonsei University College of Medicine, Seoul, 03722 South Korea

## Abstract

Non-small cell lung cancer (NSCLC) patients with EGFR mutations initially respond well to EGFR tyrosine kinase inhibitors (TKIs) but eventually exhibit acquired or innate resistance to the therapies typically due to gene mutations, such as EGFR T790M mutation or a second mutation in the downstream pathways of EGFR. Importantly, a significant portion of NSCLC patients shows TKI resistance without any known mechanisms, calling more comprehensive studies to reveal the underlying mechanisms. Here, we investigated a synthetic lethality with gefitinib using a genome-wide RNAi screen in TKI-resistant EGFR-mutant NSCLC cells, and identified RNF25 as a novel factor related to gefitinib resistance. Depletion of RNF25 expression substantially sensitized NSCLC cells to gefitinib treatment, while forced expression of RNF25 augmented gefitinib resistance in sensitive cells. We demonstrated that RNF25 mediates NF-κB activation in gefitinib-treated cells, which, in turn, induces reactivation of ERK signal to cause the drug resistance. We identified that the ERK reactivation occurs via the function of cytokines, such as IL-6, whose expression is transcriptionally induced in a gefitinib-dependent manner by RNF25-mediated NF-κB signals. These results suggest that RNF25 plays an essential role in gefitinib resistance of NSCLC by mediating cross-talk between NF-κB and ERK pathways, and provide a novel target for the combination therapy to overcome TKI resistance of NSCLC.

## Introduction

Lung cancer is the leading and the second leading cause of global cancer-related mortality of males and females, respectively^1–3^. The median survival time for patients with advanced non-small cell lung cancer (NSCLC), which accounts for about 85% of lung cancers, is less than 1 year^4, 5^. In many NSCLC patients, epidermal growth factor receptor (EGFR)-mediated cell signals are frequently upregulated due to the amplification or mutation of EGFR gene^4–6^. The two most common activating EGFR mutations are small in-frame deletions in exon 19 and amino acid substitution (L858R) in exon 21, which collectively account for about 90% of known activating EGFR mutations^7^.

NSCLC patients with EGFR mutations are responsive to first-generation EGFR inhibitors such as gefitinib and erlotinib, which have been approved by FDA as the first-line NSCLC therapies, resulting in longer median survival up to 24–30 months than those observed in patients with wild-type (WT) EGFR^8^. The higher sensitivity of cancers with these mutations is due to an increased affinity of EGFR TKIs to the ATP-binding pocket of EGFR as compared with their affinity to WT EGFR. However, in spite of the remarkably high response rates to the first-generation EGFR inhibitors, only 5% of EGFR-mutated NSCLC patients respond well and achieve tumor reduction of >90% in clinical practices^9^. In addition, these EGFR tyrosine kinase inhibitors (TKIs) have shown measurable efficacy at early stages of treatment but patients become resistant to these drugs after several months, which finally leads to treatment failure^10, 11^. Many mechanisms of either innate or acquired resistance have been discovered, including T790M mutation of EGFR, MET amplification, PTEN deletion, and a second mutation in the downstream pathway of EGFR^12–18^. Among them, the T790M mutation of EGFR is the most common cause for the resistance^16^. The second-generation EGFR TKIs, such as afatinib and dacomitinib, were developed to treat a resistant disease, targeting not only T790M, but also EGFR-activating mutations and the wild-type EGFR^19^. However, unlike the effective anti-T790M activity in the laboratory, the clinical efficacy in patients with T790M^+^ NSCLC was poor, with a response rate less than 10% among patients resistant to gefitinib or erlotinib and with dose-limiting toxicity due to simultaneous inhibition of the WT EGFR^19–21^. Recently, mutant-selective third-generation EGFR-TKIs, such as osimertinib, rociletinib, and olmutinib, which specifically and irreversibly block T790M mutant EGFR, were developed to treat EGFR T790M mutant cancers^19^.

Besides the known resistance mechanisms to EGFR TKIs, many NSCLC cancer patients exhibit innate resistance to TKIs without any known resistance mechanism. Therefore, their molecular systems of diminished response during EGFR TKI therapy, to our knowledge, are yet to be clearly understood, and additional pathways that might inhibit the growth of NSCLC with mutated EGFR need to be uncovered. Here, we investigated the synthetic lethality with gefitinib using a genome-wide RNAi screen in TKI-resistant EGFR-mutated NSCLC cells, and identified RNF25 as a factor closely related to gefitinib resistance. Depleting RNF25 expression substantially inhibited the proliferation of gefitinib-resistant NSCLC cells by inducing apoptosis through the suppression of NF-κB signaling and EGFR-independent reactivation of ERK during a prolonged drug treatment. This study provides a potential combination therapy strategy to overcome drug resistance in NSCLC based on the identification of the pathways that allow cancer cells to circumvent the primary target effects.

## Materials and methods

### Chemicals, cell Culture, DNA plasmids, small interfering RNA, and transfection of nucleic acids

The followings were suspended in dimethyl sulfoxide: gefitinib (cayman chemical, Ann Arbor, MI, USA), ERK inhibitor SCH772984 (selleckchem, Houston, TX, USA), and NF-κB inhibitor QNZ (EVP4593) (Selleckchem, Houston, TX, USA). H1650 and HCC827 lung cancer cells and 293 T cells were purchased from the American Type Culture Collection (Manassas, VA, USA). PC-9 lung cancer cells were obtained from the Public Health England (London, UK). The patient-derived gefitinib-resistant lung cancer cells, YL05 (EGFR exon19del) and YL08 (EGFR wild-type/ALK positive), were provided by Yonsei University College of Medicine, Seoul, Korea. Patient characteristic and treatments are explicated in Supplemental Table 1. Dulbecco’s modified eagle medium (DMEM) and RPMI medium 1640 were purchased from Welgene (Gyeongsan-si, Republic of Korea). Fetal bovine serum and Penicillin-Streptomycin (10,000 U/mL) were purchased from Thermo Fisher Scientific Inc. (Grand Island (NY) and Waltham (MA), USA, respectively). The cells were cultured in RPMI-1640 or DMEM medium supplemented with 10% (v/v) FBS and 1% (v/v) Penicillin-Streptomycin, and were maintained in a 5% CO_2_ incubator at 37 °C. The pcDNA™3.1/myc-His Mammalian Expression Vectors were from Thermo Fisher Scientific Inc. (Carlsbad, CA, USA). The full-sequence RNF25 gene clone was obtained from Korea Human Gene Bank in Korea Research Institute of Bioscience and Biotechnology (Daejeon, Korea). RNF25 gene-specific siRNAs (#1: GUCAUCUGCCUCUAUGGUUdTdT, #2: CCAAAACACCCUGUUACCAdTdT, and #3: CCUGUUACCACUACUUCCAdTdT), IL-6 gene-specific siRNAs (#1: GAGACAUGUAACAAGAGUAdTdT, #2: GAGUACAAAAGUCCUGAUCdTdT, and #3: CCACUGGGCACAGAACUUAdTdT), and negative control siRNA (Bioneer Inc.; Daejeon, Korea) were used for gene knockdown experiments. Fugene® HD transfection reagent (Promega, Madison, WI, USA) was used for DNA transfection and Lipofectamine® RNAiMAX reagent (Thermo Fisher Scientific, Carlsbad, CA, USA) was used for siRNA transfection.

### Pooled shRNA library module

Pooled shRNA library Human Module 1 (HM1) (Cellecta Inc., Mountain View, CA, USA) was purchased from Addgene (http://www.addgene.com). The HM1 shRNA library is composed of 27,500 shRNAs, targeting 5,000 genes involved in cell signaling (http://www.cellecta.com/index.php). The lentiviral expression vector contained a puromycin-resistance gene (PuroR), and each shRNA was linked to a unique 18-bp barcode identifiable by sequencing.

### shRNA library virus production and infection

Lentivirus was produced according to the manufacturer’s recommended protocol (Cellecta). For each 150 mm plate, 293 T cells (12.5 × 10^6^) were transfected using 60 μL of PLUS reagent and 90 μL of Lipofectamine reagent combined with 6 μg of the shRNA library plasmid pool and 30 μg of the Cellecta packaging mix (containing the psPAX2 and pMD2 plasmids that encode Gag/Pol and VSV-G, respectively). Virus was harvested at 48 h post transfection, aliquoted, and frozen at −80 °C for later use. To calculate the multiplicity of infection (MOI), graded volumes of concentrated lentivirus were added to H1650 cells and incubated at 37 °C. After 24 h, the cells were replaced with fresh medium, harvested after 48 h, and analyzed for RFP expression on a BD FACSVerse Flow Cytometer (BD Biosciences, Piscataway, NJ, USA). The volume of virus required for an MOI of 0.5 was calculated. Cells were plated for overnight, and infected with the volume of virus required for each experiment in the presence of polybrene (8 μg/mL). Medium was replaced at the following day, and stable pools of cells were selected with 1 μg/mL of puromycin.

### shRNA library screen

H1650 cells were infected with virus at an MOI of 0.5, which was sufficient to cover the library complexity with 500-fold redundancy and ensure that most cells acquired only one unique bar-coded shRNA construct. After 24 h, the infected cells were replaced with fresh medium. After 24 h, puromycin (1 μg/mL) was added to eliminate uninfected cells. After 48 h, viable puromycin-resistant cells were plated in fresh medium and the replicates of the library-transduced cells were treated with DMSO or 10 μM (IC_50_) gefitinib. Each replicate was incubated for nine days (six population doublings). After nine days, shRNA-specific barcodes were amplified by PCR from total genomic DNA isolated from each pool of surviving cells using primers that are specific for vector sequences flanking the barcode site. The Illumina-specific primers were then used for a second round of PCR, so that the amplified products could be sequenced using the Next-Generation Sequencing system (Illumina). shRNA representation in gefitinib-treated vs. DMSO-treated condition was calculated by the fold change. Significant hits were defined as those altered by 1.5-fold, and genes with at least two shRNAs in the screen were selected.

### Cell viability assay

Cells were seeded in 96-well micro titer plates (2 × 10^3^ cells per well), incubated for 24 h, and then exposed to test materials under the indicated concentration for three days. MTT solution was added to each well, and the cells were incubated in a CO_2_ incubator for additional 2 h. After removing the medium, the formazan crystals dissolved in DMSO was added, and the absorbance was measured at 570 nm in a Luminoskan Ascent Microplate Luminometer (Thermo Scientific, Waltham, MA, USA). The percentage of cell viability was expressed with respect to the percentage of untreated controls cells, which were considered as 100% viable cells. All experiments were performed independently in triplicates.

### Colony forming assay

H1650/shRNF25 or H1650/shControl cells (5 × 10^4^) were seeded in 6-well plates and treated with gefitinib or DMSO. Two weeks later, the cells were washed with PBS buffer and stained with 0.5% crystal violet in 20% methanol for 20 min. Images were captured using a LAS-4000 image system (Fujifilm Inc., Stanford, CT, USA).

### Total RNA extraction and reverse transcription-polymerase chain reaction (RT-PCR)

Total RNA was isolated from cells using the Trizol reagent (Thermo Fisher Scientific, Carlsbad, CA, USA) according to the manufacturer’s instructions. cDNA was synthesized from total RNA using reverse transcriptase (Thermo Fisher Scientific, Carlsbad, CA, USA) and target DNA sequences were amplified by PCR. Twenty-mer primers were used for polymerase chain reaction of RNF25 (forward, CGAAACCCAGAAAGCTATGC; reverse, TGTGGAGGACCTTCAACTCC), IL-6 (forward, TACCCCCAGGAGAAGATTCC; reverse, TTTTCTGCCAGTGCCTCTTT), and β-actin (forward, GGACTTCGAGCAAGAGATGG; reverse, AGCACTGTGTTGGCGTACAG). β-Actin primers were used to standardize the amount of RNA in each sample.

### Western blotting

Cells were washed with ice-cold phosphate-buffered saline (PBS) and lysed in ice-cold RIPA buffer. After centrifugation, proteins in the supernatant were separated on 12% or 10% SDS-polyacrylamide gels and then transferred to nitrocellulose membranes. Primary antibodies against pEGFR (Cell Signaling Technology, #2236), EGFR (Millipore, #06-847), RNF25 (Abcam, ab89281), pAKT (Cell Signaling Technology, #4060), AKT (Santa Cruz Biotechnology, sc-8312), pERK (Cell Signaling Technology, #9101), ERK (Cell Signaling Technology, #9102), and β-actin (Santa Cruz Biotechnology, sc-47778) were applied overnight, and then HRP-conjugated anti-mouse or anti-rabbit antibodies were applied. Antigen-antibody complexes were detected using western blotting Luminol Reagent (Pierce Biotechnology Inc., Rockford, IL, USA). Images of membranes were captured using a LAS-4000 image system (Fujifilm Inc., Stanford, CT, USA). The relative intensities of protein bands, compared with that of the respective β-actin signal, were determined by using the Multi Gauge software, version 3.0 (Fujifilm Inc.).

### Measurement of NF-κB activity

NF-κB activity was measured by performing luciferase assay using a Dual‐Luciferase Reporter Assay System (Promega, Madison, WI, USA), according to manufacturer’s instructions. Cells were co‐transfected with different combinations of luciferase reporter; 1 μg NF-κB response element and 0.25 μg Renilla. After 24 h, the cells were treated with DMSO or gefitinib for additional 24 h. Luciferase activities were determined using Luminoskan Ascent luminometer (Thermo Scientific, Waltham, MA, USA). Data were normalized with Renilla luciferase activity. The experiments were performed independently in triplicates.

### In vivo evaluation of anticancer activity in a H1650 xenograft model

H1650/pLKO and H1650/shRNF25 cells (each 5 × 10^6^) resuspended in PBS were subcutaneously injected into the right rear flank of six week-old female BALB/c nude mice (*n* = 12/cell line) (SLC Inc., Hamamatsu, Shizuoka, Japan). Each group was randomly stratified into two subgroups and treatments were initiated when all mice had established mean tumor size about 100 mm^3^. Each subgroup was administered intraperitoneally with DMSO (10% DMSO + 5% tween 80 in PBS) or gefitinib (50 mg/kg, 10% DMSO + 5% tween 80 in PBS) every three days for six weeks. The body weight and tumor size of individual mice were recorded twice a week. The size of tumors was measured in two dimensions with a caliper. The tumor volume was calculated using the equation $\left( {2 \times l \times w} \right)/2$, where *l* and *w* represent the largest and smallest dimensions in each measurement. At the end of the study, tumor tissues were excised and snap-frozen in liquid nitrogen for biomarker analysis.

### Statistical analysis

The results are represented as mean ± S.D. *p*-values for determining statistical significance were calculated using an unpaired two-tailed Student’s *t*-test. Symbols used: *p < 0.05; **p < 0.01; ns, not significant.

## Results

### RNAi screen to identify genes related to gefitinib resistance in NSCLC

**Fig. 1:**
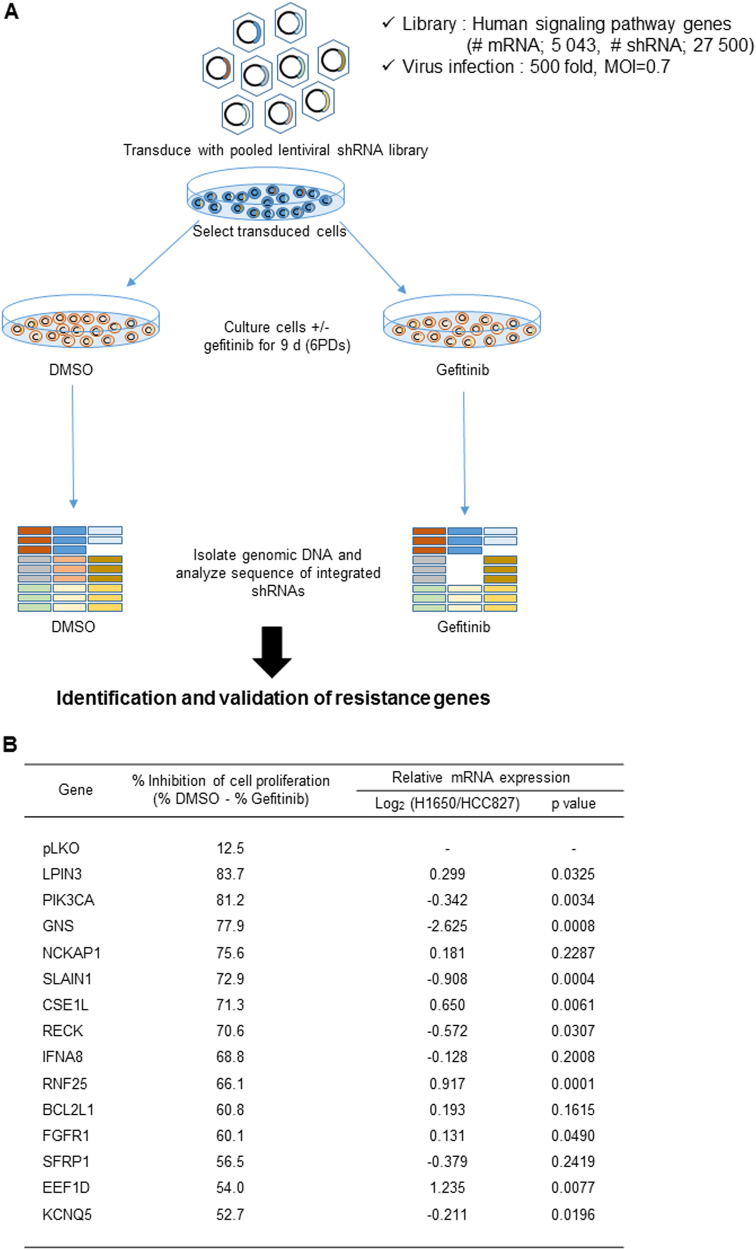
Identification of synthetic lethality genes with gefitinib in NSCLC cells using a pooled human shRNA library. **a** Scheme of the shRNA library screening. H1650 cells infected with the shRNA library were cultured with or without gefitinib (10 μM) for nine days (six passage divisions), and their genomic DNA was analyzed by sequencing to count integrated shRNAs. **b** Top-ranked candidate genes selected from the shRNA library screen showing >50% inhibition effects of synthetic lethality with gefitinib, and their relative expression in gefitinib-resistant (H1650) vs. -sensitive (HCC827) NSCLC cell lines (Log_2_ (fold change))

Among different human NSCLC cell lines carrying activating EGFR mutations, H1650 exhibited the most prominent resistance to gefitinib treatment^22^. H1650 cells additionally harbor PTEN loss, which can also mediate resistance to EGFR TKIs^17^. However, given that loss of PTEN did not fully account for their drug insensitivity, we decided to use H1650 for the loss-of-function genetic screen, expecting identification of novel genes involved in gefitinib resistance of NSCLC. A pool of viruses harboring a human shRNA library that collectively target 5,043 genes curated to major canonical and non-canonical pathways, covered by KEGG, Reactome, and other expert-curated pathway databases, was transduced into H1650 cells. The cells were then treated with DMSO or 10 μM gefitinib, a dose that did not substantially impair the proliferation of parental H1650 cells. After six rounds of population doubling, changes in the shRNA species represented in the surviving cell populations were determined by sequencing the barcodes tagged at each shRNA construct (Fig. 1a). The normalized reads per shRNA at the end of the experiment (DMSO and Gefitinib), as well as those at the beginning of the experiment (T0) were highly correlative between duplicates (Supplemental Fig. 1A), demonstrating the reproducibility of the screening.

To identify candidate genes possibly involved in gefitinib resistance, we first sorted out the shRNA constructs selectively depleted more than 1.5-fold in gefitinib-treated cells compared to DMSO-treated cells in both duplicates. Among the genes corresponding to these shRNAs, 62 were targeted by more than two shRNAs (Supplemental Table 2). To validate the screening results, we depleted the expression of individual genes with a pool of 4–5 different shRNA constructs and determined the effect of gene depletion on cell growth by counting viable cells at 72 h after treating with gefitinib. Among the 62 candidates tested, depletion of 14 genes resulted in more than 50% inhibition of cell proliferation in the presence of 10 μM gefitinib, a dose that reduced the proliferation rate of control cells by 12.5% (Fig. 1b, Supplemental Fig. 1b). Three of the 14 selected genes, PIK3CA^23^, BCL2L1^24^, and FGFR1^25^ were previously shown as the genetic factors involved in gefitinib resistance mechanisms, serving as positive controls for the other 11 candidates in supporting their potential roles in the induction of gefitinib resistance. We then examined the mRNA expression of these genes in H1650 cells in comparison with that in a gefitinib-sensitive EGFR-mutant NSCLC cell line, HCC827, and found that RNF25 and EEF1D are expressed at more than 1.5-fold higher levels in H1650 than in HCC827 (Fig. 1b, Supplemental Fig. 1c). EEF1D (Elongation factor 1-delta) is a subunit of the elongation factor-1 complex where it functions as the guanine nucleotide exchange factor during the enzymatic delivery of aminoacyl tRNAs to ribosome^26^. RNF25, on the other hand, is an E3 ubiquitin-protein ligase with a RING finger motif, and is known to interact with RelA, the p65 subunit of NF-κB to modulate its transcriptional activity^27^. NF-κB signaling plays crucial roles in tumorigenesis by providing pro-survival signals, as well as inflammatory microenvironment to tumor cells^28^. Therefore, in our subsequent studies, we focused on RNF25 to elucidate its possible roles in mediating the gefitinib resistance in NSCLC.

### RNF25 regulates ERK reactivation responsible for the gefitinib resistance

**Fig. 2:**
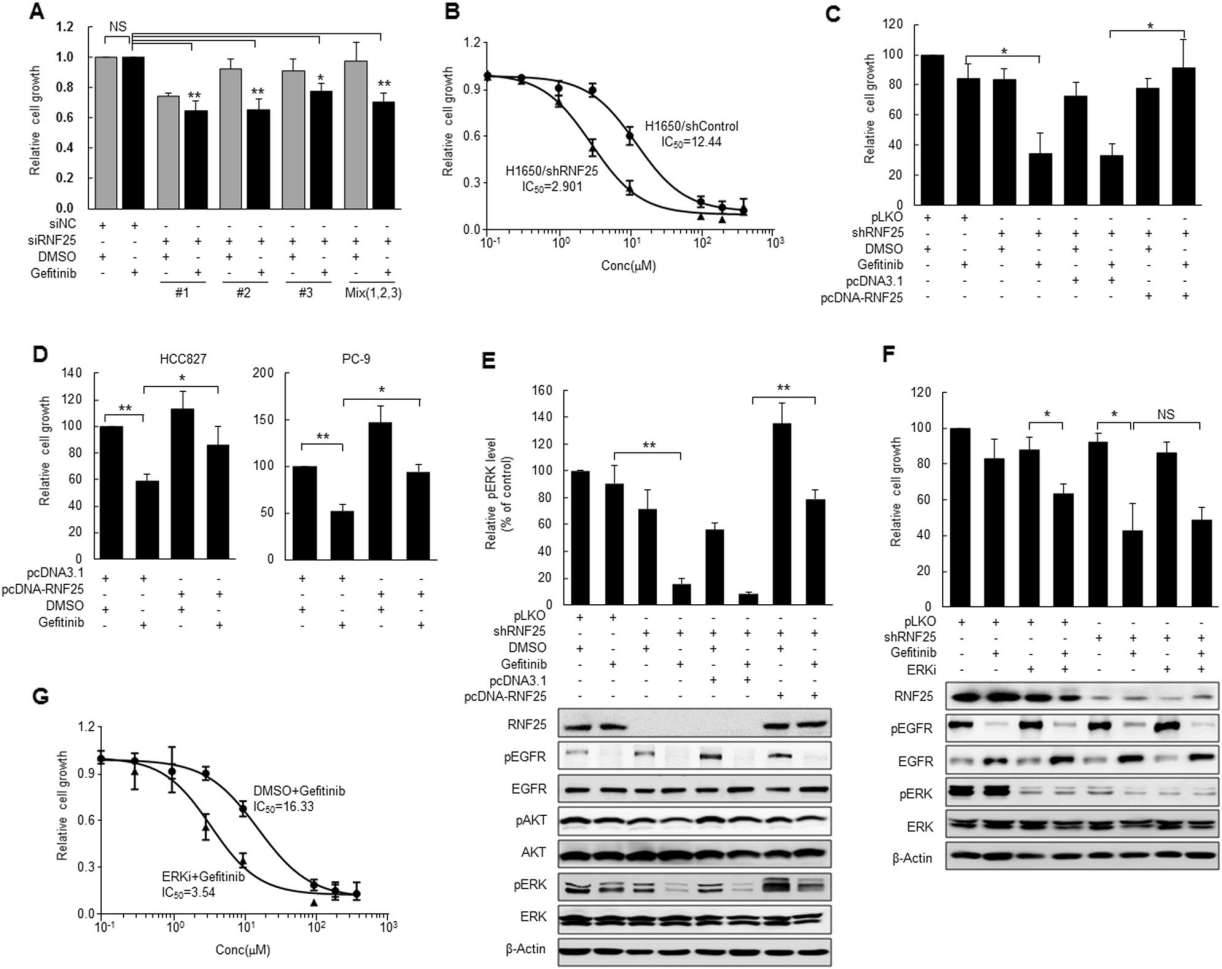
RNF25 knockdown increases gefitinib sensitivity by inhibiting ERK reactivation in gefitinib-resistant cells. **a** H1650 cells were transfected with siRNAs for RNF25 individually or as a mixture, and then treated with gefitinib (5 μM) or DMSO for 72 h. Cell proliferation was measured by cell counting. Values are mean ± SD of three independent experiments. **b**, **c** H1650 cells stably transduced with a lentiviral vector carrying shRNF25 (H1650/shRNF25) or pLKO (H1650/shControl) were treated with gefitinib or DMSO for 72 h. The gefitinib sensitivity of each cell line was determined using varying concentrations of gefitinib, and the values are mean ± SD of two independent experiments (**b**) For reconstitution of RNF25 expression, H1650/shRNF25 (**c**). **d** HCC827 or PC-9 cells were transfected with pcDNA-RNF25 or a control vector for 24 h, and then treated with gefitinib (5 μM for H1650, 0.002 μM for HCC827, 0.05 μM for PC-9). For (**c**, **d**), values are mean ± SD of three independent experiments. **e**, **f** Phosphorylation levels of pro-survival pathway members were analyzed by western blotting in H1650 cells stably transduced with a lentiviral vector carrying shRNF25 or pLKO. The cells were treated with gefitinib (5 μM), ERK inhibitor (ERKi; SCH772984, 10 μM), and/or DMSO for 72 h before the western blot analysis. Values in the bar graphs are mean ± SD of three independent experiments. β-Actin was used as a loading control. **g** H1650 cells were treated with ERK inhibitor (10 μM) or vehicle in combination with varying concentrations of gefitinib. Values are mean ± SD of two independent experiments. Statistical significance was determined by the Student’s *t*-test (*p < 0.05 and **p < 0.01)

The involvement of RNF25 in gefitinib resistance of H1650 cells was confirmed using three different siRNAs targeting RNF25 or their mixture. Depletion of RNF25 in conjunction with gefitinib treatment significantly reduced the cell proliferation whereas either RNF25 loss or gefitinib treatment alone had only marginal effects (Fig. 2a, Supplemental Fig. 2A). In addition, H1650 cells stably transduced with an RNF25-specific shRNA (H1650/shRNF25) exhibited an increased response to gefitinib with an IC_50_ value about 4.3-fold lower than that of the control shRNA-transduced cells (H1650/shControl) (Fig. 2b, Supplemental Fig. 2B). Reconstitution of RNF25 expression in H1650/shRNF25 cells, where RNF25 had been depleted by an shRNA targeting its 3′ UTR sequences restored the cell proliferation in the presence of gefitinib, further confirming the role of RNF25 in the induction of gefitinib resistance (Fig. 2c, Supplemental Fig. 2C). Moreover, forced expression of exogenous RNF25 in gefitinib-sensitive HCC827 or PC-9 cells desensitized them to gefitinib (Fig. 2d, Supplemental Figs. 1C and 2D).

We investigated the molecular mechanism underlying RNF25-mediated gefitinib resistance in H1650 cells by examining the activity of pro-survival signaling pathways at 72 h after gefitinib treatment. Treatment with 5 μM gefitinib effectively inactivated phosphorylation of EGFR in H1650, but failed to decrease the phosphorylation of AKT and ERK (Fig. 2e). RNF25 loss by itself did not affect the phosphorylation status of these three pro-survival pathway members. In contrast, gefitinib treatment clearly decreased the ERK phosphorylation level in H1650/shRNF25 cells, while this inhibition was rescued by restoration of RNF25 expression (Fig. 2e). Inactivation of EGFR by TKIs, such as erlotinib leads to acute reduction in phosphorylated ERK level^29^. However, the ERK signaling often becomes reactivated, restoring the ERK phosphorylation level in TKI-treated cells^14, 30^. The inactivation and reactivation of ERK signaling were also observed in gefitinib-treated H1650 cells (Supplemental Fig. 3). These results suggest that RNF25 is involved in the reactivation of ERK pathway in gefitinib-treated cells.

We then examined if targeting the ERK signaling, whose reactivation was dependent on RNF25, could sensitize H1650 cells to gefitinib treatment. Combinatorial treatment of the ERK inhibitor SCH772984 (10 μM) along with gefitinib (5 μM) inactivated both EGFR and ERK signals in H1650 cells and decreased their proliferation to a level comparable to that of RNF25-depleted cells treated with gefitinib (Fig. 2f, g). By contrast, combinatorial effect of SCH772984 and gefitinib was not observed in RNF25-depleted cells (Fig. 2f). Together, these results suggest that RNF25 is intimately involved in the reactivation of ERK pathway that confers gefitinib resistance to H1650 cells.

### RNF25-mediated induction of NF-κB signaling is responsible for the ERK reactivation and gefitinib resistance

**Fig. 3:**
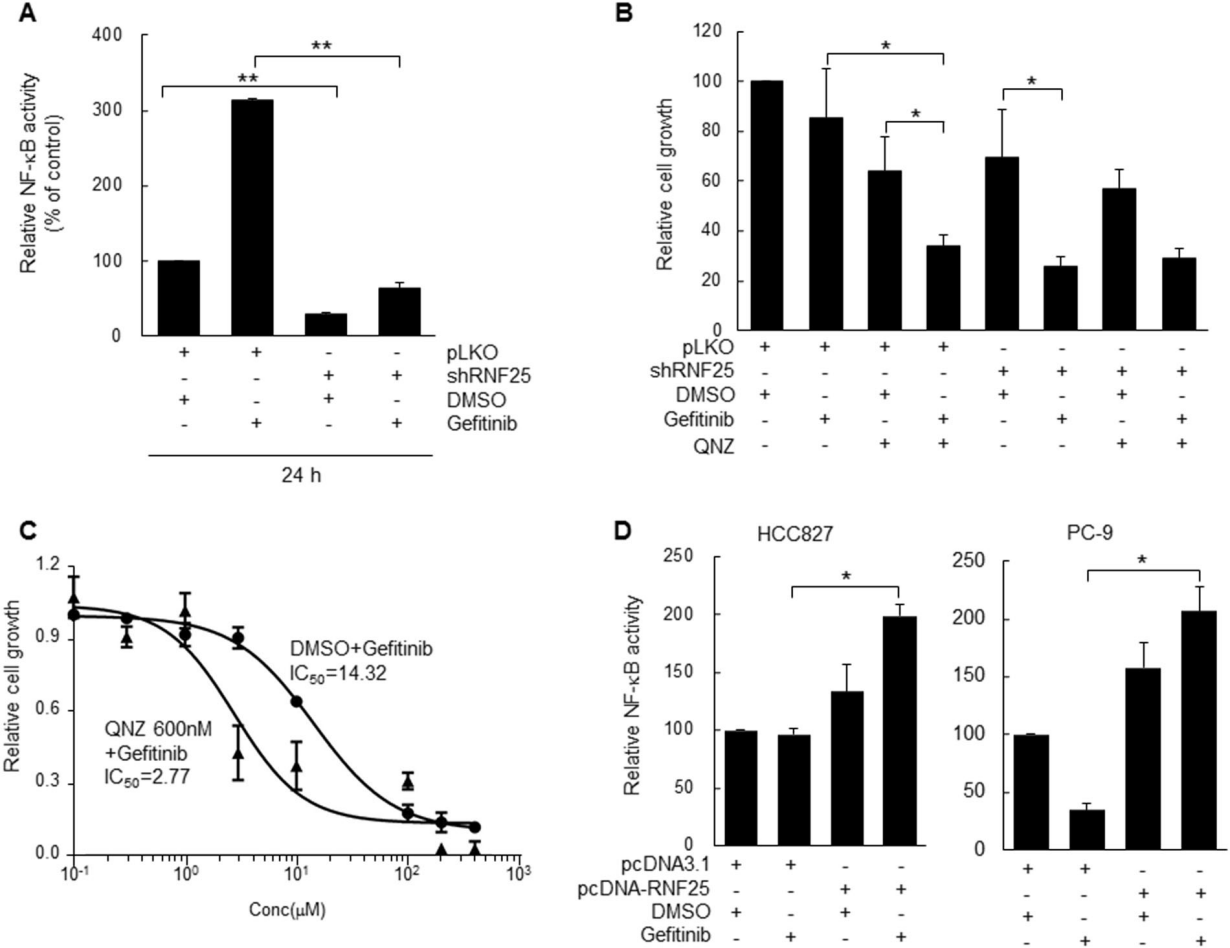
Downregulation of NF-κB signaling by RNF25 knockdown increases gefitinib sensitivity. **a** H1650/shRNF25 or H1650/shControl cells transfected with NF-κB luciferase reporter plasmid were treated with gefitinib (5 μM) or DMSO for 24 h, and then determined for NF-κB activity. Values are mean ± SD of three independent experiments. **b**, **c** H1650/shRNF25 or H1650/shControl cells were pretreated with an NF-κB inhibitor (QNZ, 600 nM) for 2 h, and then treated with 5 μM (**b**) or varying concentrations (**c**) of gefitinib. Cell proliferation was measured by cell counting. Values are mean ± SD of three (**b**) to two (**c**) independent experiments. **d** HCC827 or PC-9 cells transduced with pcDNA-RNF25 or pLKO vector were treated with gefitinib (0.002 μM for HCC827, 0.05 μM for PC-9), and then determined for NF-κB activity. Values are mean ± SD of three independent experiments. Statistical significance was determined by the Student’s *t*-test (*p < 0.05 and **p < 0.01)

It is known that RNF25 promotes NF-κB-mediated transcription by interacting with the p65 subunit of NF-κB^15, 27^. Deregulated NF-κB signals can be pro-tumorigenic by providing survival signals and favorable tumor microenvironment to tumor cells^28^. We, therefore, examined whether the gefitinib resistance of H1650 cells is associated with the RNF25-dependent changes in NF-κB signaling activities. When H1650 cells were treated with gefitinib, NF-κB activity was highly augmented (Supplemental Fig. 4). However, depletion of RNF25 abolished both the gefitinib-induced, as well as the basal-level NF-κB activities (Fig. 3a). This result demonstrates that RNF25 plays a crucial role in the activation of NF-κB signaling in H1650 cells. Consistently, co-treatment with an NF-κB inhibitor, QNZ, along with gefitinib significantly aggravated the proliferation deficit of H1650 cells compared to the treatment of either agent alone (Fig. 3b). When combined with 600 nM QNZ, the IC_50_ value of gefitinib in H1650 cells decreased about 5.2-fold compared to that without QNZ, similar to the change in IC_50_ value when gefitinib treatment was combined with RNF25 knockdown (Figs. 3c and 2b). However, QNZ treatment failed to further decrease the proliferation of RNF25-depleted cells that were treated with gefitinib (Fig. 3b). On the other hand, gefitinib did not increase NF-κB activity in both HCC827 and PC-9 cells, while RNF25 overexpression caused a gefitinib-induced increase in NF-κB activity in these cells (Fig. 3d). These results suggest that RNF25 confers gefitinib resistance to EGFR-mutant lung cancer cells by mediating gefitinib-dependent induction of NF-κB signaling, while its loss sensitizes the gefitinib-resistant cells to the drug by suppressing the induction of NF-κB signaling.

**Fig. 4:**
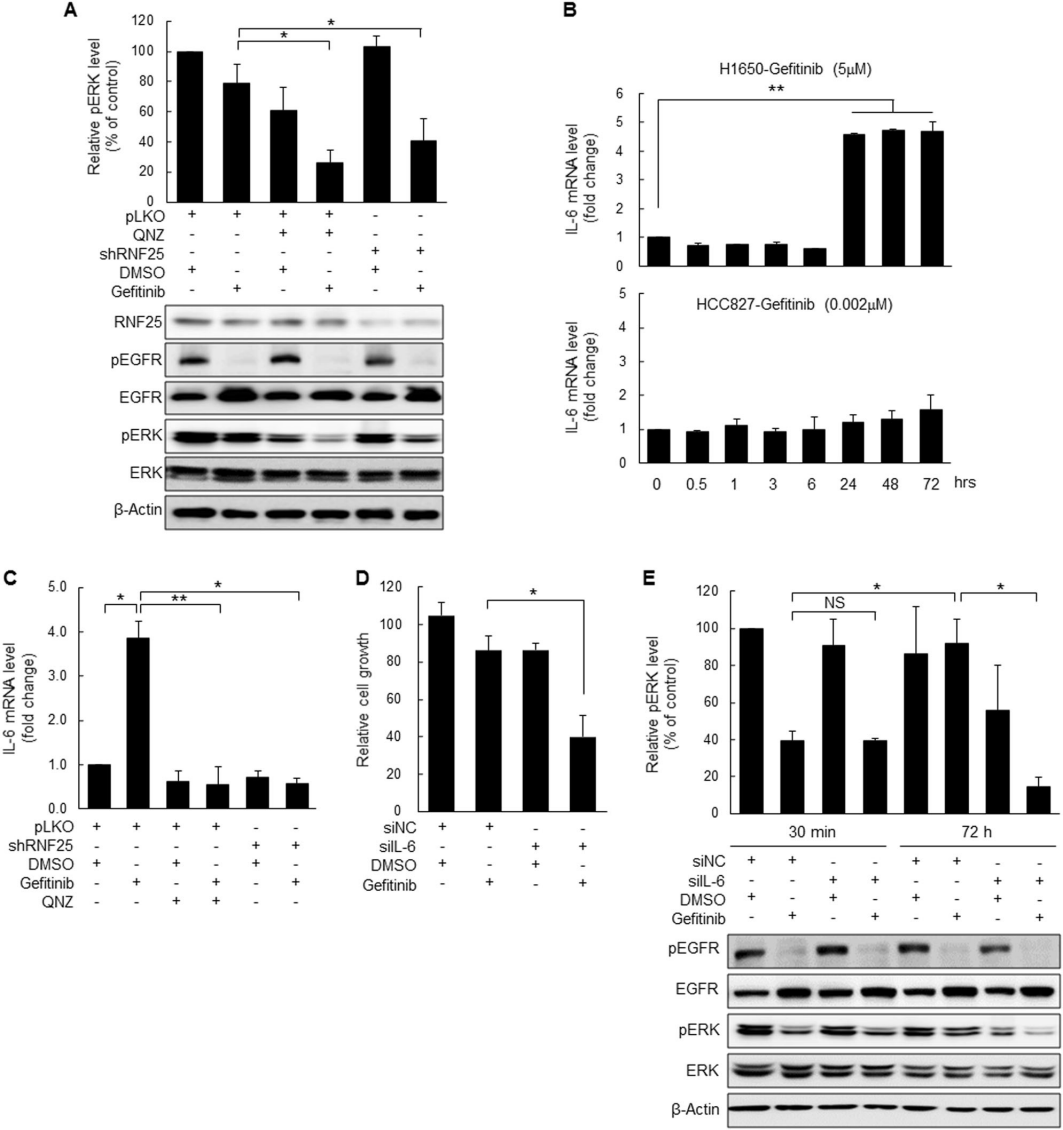
RNF25-induced NF-κB signals are responsible for the ERK reactivation that confers gefitinib resistance to EGFR-mutant NSCLC cells. **a** H1650/shRNF25 or H1650/shControl cells were treated with an NF-κB inhibitor (QNZ, 600 nM) and the levels of p-EGFR and p-ERK were determined by western blotting. Values are mean ± SD of three independent experiments. **b**, **c** Expression of IL-6 mRNA was determined by real-time RT-PCR in H1650 or HCC827 cells at the indicated time points (**b**), or in H1650/shRNF25 or H1650/shControl cells at 48 h (**c**) after treatment with gefitinib (5 μM). Values are mean ± SD of two (**b**) to three (**c**) independent experiments. **d** Silencing of IL-6 expression sensitized the H1650 cells to gefitinib. Values are mean ± SD of three independent experiments. **e** Silencing of IL-6 expression suppressed ERK reactivation in gefitinib-treated cells. Values are mean ± SD of three independent experiments. Statistical significance was determined by the Student’s *t*-test (*p < 0.05 and **p < 0.01)

Given that the activities of both NF-κB and ERK signals in gefitinib-treated H1650 cells are intimately related to RNF25 expression, we examined whether the two signals cross-talk with each other via an RNF25-mediated process. We, therefore, measured NF-κB-dependent changes in p-ERK level in gefitinib-treated H1650 cells and observed a clear suppression of the ERK reactivation by QNZ (Fig. 4a), which was quantitatively comparable to the suppression of ERK reactivation by RNF25 depletion. These results imply that RNF25-mediated NF-κB signal activation is closely linked to the ERK reactivation in gefitinib-treated cells. In order to identify the link between the two events, we examined the role of NF-κB targets whose expression is upregulated in a gefitinib-dependent manner in H1650 cells while also activating MAPK signaling. Pro-inflammatory cytokines, such as IL-1β and IL-6, which are the transcriptional targets of NF-κB signals, can activate MAPK pathway signals^31–35^. In particular, IL-6 functions as the pro-survival factor in addition to its dominant roles in inflammatory responses^31^. We checked the time course of IL-6 mRNA expression following gefitinib treatment in H1650 cells, and found a clear induction of IL-6 expression at around the time when the gefitinib-induced ERK reactivation was kicked off (Fig. 4b, Supplemental Fig. 3). In contrast, the gefitinib-mediated induction of IL-6 expression was not observed in HCC827 cells even when they were treated with the drug at twice the IC_50_ concentration (Supplemental Fig. 1C). The gefitinib-mediated IL-6 induction was dependent on RNF25 and NF-κB signal since it was abrogated by NF-κB inhibitor treatment or RNF25 knockdown (Fig. 4c). Furthermore, we found that loss of IL-6 made H1650/sh control cells sensitive to gefitinib to the level comparable to that of RNF25 depletion (Fig. 4d). We also found that gefitinib-induced ERK reactivation is definitely suppressed by IL-6 depletion in H1650 cells, similar to RNF25 depletion (Fig. 4e). These results suggest that RNF25 is a major player in connecting NF-κB signaling to ERK signal reactivation in gefitinib-treated cells via the induction of ERK-activating cytokine expression.

### Loss of RNF25 sensitizes EGFR-mutant NSCLC cells to gefitinib in vivo

**Fig. 5:**
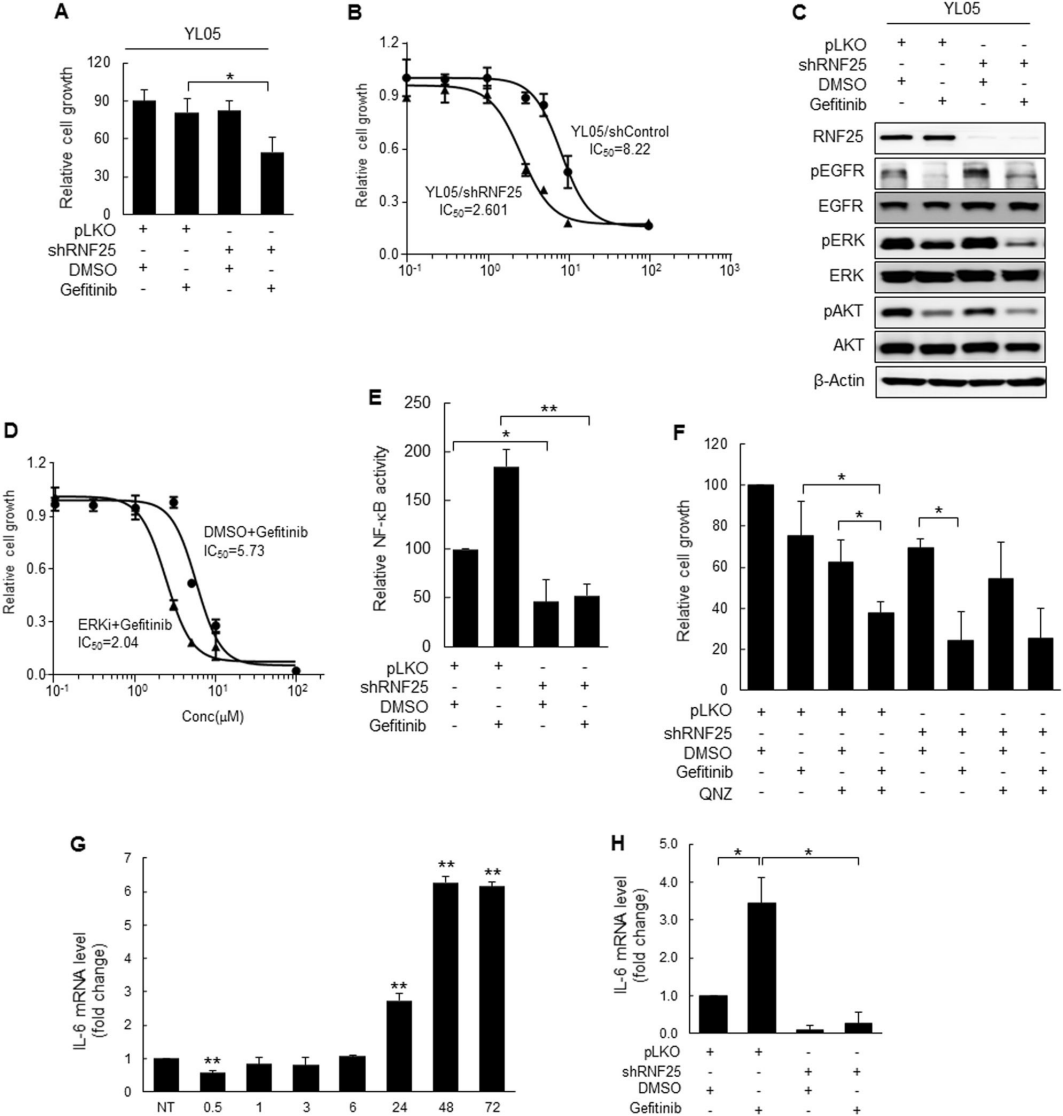
RNF25 depletion sensitizes gefitinib-resistant patient-derived cells (PDCs) to gefitinib. **a**, **b** Gefitinib-resistant lung cancer PDC YL05 cells stably transduced with a lentiviral vector carrying shRNF25 (YL05/shRNF25) or pLKO (YL05/shControl) were treated with gefitinib at 5 uM (**a**) or various concentrations (**b**) for three days. Cell proliferation was measured by cell counting. **c** YL05/shRNF25 or YL05/shControl cells were treated with gefitinib (5 μM) or DMSO for three days. Protein levels were analyzed by western blotting. β-Actin was used as a loading control. **d** Gefitinib-resistant YL05 PDCs were treated with gefitinib (5 μM) only (DMSO + Gefitinib) or together with ERK inhibitor (10 μM) (ERKi + Gefitinib) for three days. **e** YL05/shRNF25 or YL05/shControl cells transfected with NF-κB luciferase reporter plasmid were treated with gefitinib (5 μM) or DMSO for 48 h, and then determined for NF-κB activity. **f** YL05/shRNF25 or YL05/shControl cells were pretreated with an NF-κB inhibitor (QNZ, 600 nM) for 2 h, and then treated with 5 μM of gefitinib for 3 days. **g**, **h** Expression of IL-6 mRNA was determined by real-time RT-PCR in YL05 cells at the indicated time points (**g**), or in YL05/shRNF25 or YL05/shControl cells at 72 h (**h**) after treatment with gefitinib (5 μM). Values are mean ± SD of three independent experiments. Statistical significance was determined by the Student’s *t*-test (*p < 0.05 and **p < 0.01)

The involvement of RNF25 in gefitinib resistance was further validated by employing cell lines derived from tumors of lung cancer patients (PDCs) showing gefitinib resistance. Consistent with the results obtained from H1650 cells, depletion of RNF25 in conjunction with gefitinib treatment significantly reduced proliferation of YL05 (EGFR exon19del) cells, whereas either RNF25 loss or gefitinib treatment alone had only marginal effects (Fig. 5a). In addition, YL05 cells stably transduced with an RNF25-specific shRNA (YL05/shRNF25) exhibited an increased response to gefitinib with an IC_50_ value about 3.2-fold lower than that of the control shRNA-transduced cells (YL05/shControl) (Fig. 5b). ERK signaling was also reactivated at a later point in time in gefitinib-treated YL05 cells (Supplemental Fig. 5a) and the gefitinib-induced ERK reactivation was abrogated in the RNF25-depleted YL05 (carrying mutant EGFR) (Fig. 5c) and even YL08 (carrying wild-type EGFR) PDCs (Supplemental Fig. 5B). Combinatorial treatment of the ERK inhibitor SCH772984 (10 μM) along with gefitinib (5 μM) increased response to gefitinib with an IC_50_ value about 2.8-fold lower than that of the YL05 cells treated with gefitinib only (Fig. 5d). Next, we validated that NF-κB signaling is induced in gefitinib-treated YL05 cells and that RNF25 plays a crucial role in the activation of NF-κB signaling in YL05 cells. NF-κB activity was augmented by gefitinib treatment, but abolished by depletion of RNF25 in gefitinib-treated YL05 cells (Fig. 5e). Consistently, co-treatment with an NF-κB inhibitor, QNZ, along with gefitinib significantly aggravated the proliferation deficit of the cells compared to the treatment of either agent alone in YL05 cells, but not in the RNF25-depleted YL05 cells (Fig. 5f). Lastly, we confirmed that IL-6 expression was clearly inducted at around the time when the gefitinib-induced ERK reactivation was kicked off (Fig. 5g), and that the induced expression was abrogated by RNF25 knockdown (Fig. 5h). Furthermore, RNF25 depletion significantly decreased both anchorage-dependent and -independent growth of the patient-derived both cells (YL05(EGFR exon19del) and YL08 (wild-type EGFR) PDCs) treated with 5 μM gefitinib (Supplemental Fig. 5c (colony formation assay), and 5D (anchorage-independent growth assay in soft agar)). Together, these suggests the clinical relevance of RNF25/NF-κB/ERK axis in the induction of TKI resistance in lung cancer cells.

**Fig. 6:**
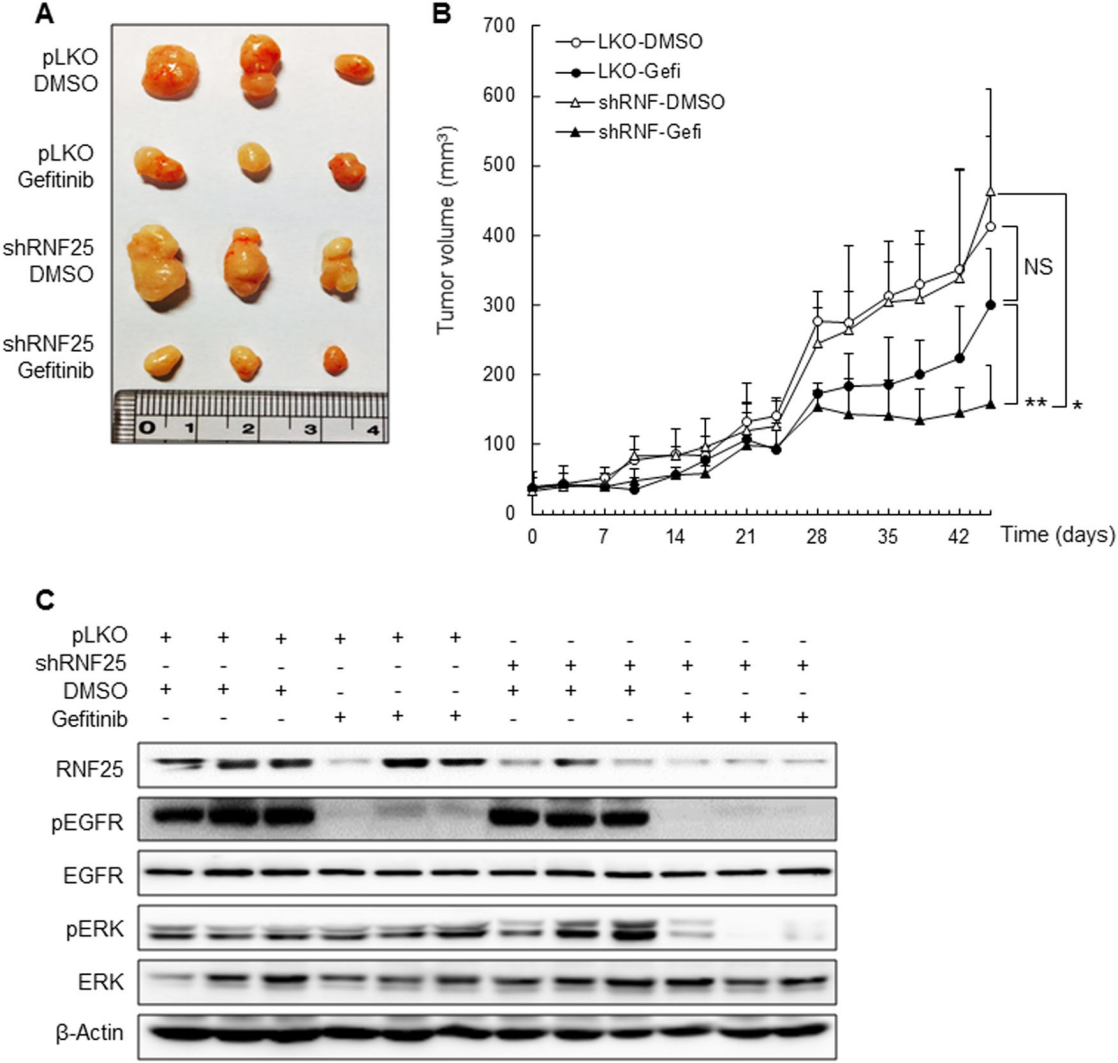
The effects of altered RNF25 expression on the anti-tumor efficacy of gefitinib in vivo. **a**, **b** Mice bearing H1650/shRNF25 or H1650/shControl tumor xenografts were injected twice a week with 50 mg/kg gefitinib or vehicle by i.p., and tumor growth was monitored for 45 days. Photographs of tumors resected from each group at the end of experiments are shown (**a**) *n* = 3/group. Profiles of tumor growth during the 45 days of gefitinib treatment are also shown; For each treatment group, data are presented as mean tumor volume (mm^3^) ± SEM (**b**). **c** The levels of protein expression in the resected tumors were analyzed by western blotting. β-Actin was used as a loading control

Finally, we examined whether the suppression of gefitinib resistance by RNF25 depletion in vitro is recapitulated in vivo using H1650 cells xenografted to nude mice. We generated mice harboring H1650-derived tumors expressing shRNF or pLKO, and treated them twice a week with an intraperitoneal injection of gefitinib (50 mg/kg) or vehicle. In parallel with the in vitro results, RNF25 knockdown alone did not affect tumor growth in the vehicle-treated group (Fig. 6a, b). However, gefitinib treatment in combination with RNF25 depletion significantly inhibited tumor growth compared to the treatment with either alone. Western blot analysis of resected tumors indicated that ERK reactivation was effectively suppressed only in the group where gefitinib treatment was combined with RNF25 depletion (Fig. 6c). These in vivo data further support a role for RNF25 as a potential target to prevent resistance to gefitinib in EGFR-mutant NSCLC.

## Discussion

Nearly all NSCLC patients with either an exon 19 deletion or an exon 21 mutation in the EGFR gene exhibit an initial response to TKI therapy, but the tumors invariably become resistant to therapy after 9–14 months^36^. A number of resistance mechanisms have been identified, including acquisition of EGFR-T790M mutations, insertion mutations in exon 20 of EGFR gene, PIK3CA mutations, and amplification of MET or FGFR1^16, 37, 38^. However, the resistance mechanisms are unknown in a large proportion of TKI-resistant patients, calling more efforts to disclose more comprehensive resistance mechanisms. In this study, to identify genes whose depletion results in synthetic lethality with gefitinib, we performed a loss-of-function screen assay in an EGFR-mutant NSCLC cell line. Among the genes identified, we selected RNF25 as a novel gene involved in gefitinib resistance and investigated its role and molecular mechanism of action in inducing the drug resistance.

RNF25, a RING finger-dependent E3 ubiquitin ligase, participates in NF-κB signaling, which regulates the expression of many cytokines involved in cell proliferation, survival, and inflammation^27, 39, 40^. Cytokines can induce MEK/ERK signals via the receptors, such as gp130 and CXCR1/2^41, 42^. Accumulating evidence has demonstrated that both NF-κB and ERK signals are implicated in the induction of drug resistance in TKI-resistant NSCLC cells^14, 15^. For example, the level of cytokines, including IL-6 and IL-8, was upregulated in TKI-resistant cells and in the plasma of TKI-resistant cancer patients^43, 44^. In addition, overexpression of the cytokines suppressed gefitinib-induced apoptosis in gefitinib-sensitive cells, while their suppression enhanced gefitinib-induced cell death in gefitinib-resistant cells^43, 44^. These previous reports are consistent with our findings that the RNF25-dependent upregulation of NF-κB activity in response to gefitinib treatment is closely related to the reactivation of ERK signaling and the induction of gefitinib resistance. We showed that RNF25 depletion suppressed the NF-κB activation and, as the consequence, blocked the reactivation of ERK signaling in gefitinib-treated cells. This effect of RNF25 depletion on ERK reactivation was closely associated with the suppression of IL-6 expression. Therefore, we reason that RNF25 functions as an essential mediator connecting NF-κB and ERK pathways, and its depletion disrupts this connection, thereby sensitizing the EGFR-mutant NSCLC cells to gefitinib treatment.

Expression of RNF25 is elevated in gefitinib-resistant EGFR-mutant cell line H1650 and gefitinib-resistant PDCs. Depletion of RNF25 prominently impaired the proliferation of gefitinib-resistant NSCLC PDCs, as well as H1650 cells in the presence of gefitinib, while augmentation of RNF25 expression in gefitinib-sensitive EGFR-mutant cells rendered them more refractory to gefitinib treatment. H1650 NSCLC cells were shown to exhibit resistance to other TKIs, too, including erlotinib and afatinib^11, 45^. Although H1650 cells harbor a known EGFR TKI resistance mechanisms, i.e., functional PTEN loss, it does not fully account for their insensitivity to TKIs. We postulate that the RNF25 expression level may be related to general TKI resistance. Supporting it, other mutant EGFR TKIs, erlotinib and afatinib, also stimulated an NF-κB-mediated transcriptional survival program in H1650 NSCLC cells^46^. In addition, we observed that RNF25 level is increased in the cells showing resistance to either erlotinib or ALK inhibitor (data not shown).

Therefore, our study provides a theoretical basis for overcoming TKI resistance in NSCLC by identifying RNF25 as a clinical target for the combination therapy of NSCLC cells that harbor activating EGFR mutations.

## Electronic supplementary material

Supplemental Table 1

Supplemental Table 2

Supplemental Figure Legends

Supplemental Figure 1

Supplemental Figure 2

Supplemental Figure 3

Supplemental Figure 4

Supplemental Figure 5

## References

[1] Pao W, Chmielecki J (2010). Rational, biologically based treatment of EGFR-mutant non-small-cell lung cancer. Nat. Rev. Cancer.

[2] Chong CR, Janne PA (2013). The quest to overcome resistance to EGFR-targeted therapies in cancer. Nat. Med..

[3] Torre LA (2015). Global cancer statistics, 2012. CA Cancer J. Clin..

[4] Herbst RS, Heymach JV, Lippman SM (2008). Lung cancer. N. Engl. J. Med..

[5] Schiller JH (2002). Comparison of four chemotherapy regimens for advanced non-small-cell lung cancer. N. Engl. J. Med..

[6] Phuchareon J, McCormick F, Eisele DW, Tetsu O (2015). EGFR inhibition evokes innate drug resistance in lung cancer cells by preventing Akt activity and thus inactivating Ets-1 function. Proc. Natl Acad. Sci. USA.

[7] Li AR (2008). EGFR mutations in lung adenocarcinomas: clinical testing experience and relationship to EGFR gene copy number and immunohistochemical expression. J. Mol. Diagn..

[8] Wu K (2013). Gefitinib resistance resulted from STAT3-mediated Akt activation in lung cancer cells. Oncotarget.

[9] Rosell R (2012). Erlotinib versus standard chemotherapy as first-line treatment for European patients with advanced EGFR mutation-positive non-small-cell lung cancer (EURTAC): a multicentre, open-label, randomised phase 3 trial. Lancet Oncol..

[10] Morris LG, Chan TA (2011). Resistance to EGFR inhibitors: molecular determinants and the enigma of head and neck cancer. Oncotarget.

[11] Chin TM (2008). Reduced Erlotinib sensitivity of epidermal growth factor receptor-mutant non-small cell lung cancer following cisplatin exposure: a cell culture model of second-line erlotinib treatment. Clin. Cancer Res..

[12] Yamasaki F (2007). Acquired resistance to erlotinib in A-431 epidermoid cancer cells requires down-regulation of MMAC1/PTEN and up-regulation of phosphorylated Akt. Cancer Res..

[13] Kokubo Y (2005). Reduction of PTEN protein and loss of epidermal growth factor receptor gene mutation in lung cancer with natural resistance to gefitinib (IRESSA). Br. J. Cancer.

[14] Ercan D (2012). Reactivation of ERK signaling causes resistance to EGFR kinase inhibitors. Cancer Discov..

[15] Bivona TG (2011). FAS and NF-kappaB signalling modulate dependence of lung cancers on mutant EGFR. Nature.

[16] Onitsuka T (2010). Acquired resistance to gefitinib: the contribution of mechanisms other than the T790M, MET, and HGF status. Lung Cancer.

[17] Sos ML (2009). PTEN loss contributes to erlotinib resistance in EGFR-mutant lung cancer by activation of Akt and EGFR. Cancer Res..

[18] Engelman JA (2007). MET amplification leads to gefitinib resistance in lung cancer by activating ERBB3 signaling. Science.

[19] Sullivan I, Planchard D (2016). Next-generation EGFR tyrosine kinase inhibitors for treating EGFR-mutant lung cancer beyond first line. Front Med (Lausanne).

[20] Miller VA (2012). Afatinib versus placebo for patients with advanced, metastatic non-small-cell lung cancer after failure of erlotinib, gefitinib, or both, and one or two lines of chemotherapy (LUX-Lung 1): a phase 2b/3 randomised trial. Lancet Oncol..

[21] Reckamp KL (2014). A phase 2 trial of dacomitinib (PF-00299804), an oral, irreversible pan-HER (human epidermal growth factor receptor) inhibitor, in patients with advanced non-small cell lung cancer after failure of prior chemotherapy and erlotinib. Cancer.

[22] Sudo M (2013). Inhibiting proliferation of gefitinib-resistant, non-small cell lung cancer. Cancer Chemother. Pharmacol..

[23] Engelman JA (2006). Allelic dilution obscures detection of a biologically significant resistance mutation in EGFR-amplified lung cancer. J. Clin. Invest..

[24] Hata AN (2016). Tumor cells can follow distinct evolutionary paths to become resistant to epidermal growth factor receptor inhibition. Nat. Med..

[25] Terai H (2013). Activation of the FGF2-FGFR1 autocrine pathway: a novel mechanism of acquired resistance to gefitinib in NSCLC. Mol. Cancer Res..

[26] Sivan G, Aviner R, Elroy-Stein O (2011). Mitotic modulation of translation elongation factor 1 leads to hindered tRNA delivery to ribosomes. J. Biol. Chem..

[27] Asamitsu K, Tetsuka T, Kanazawa S, Okamoto T (2003). RING finger protein AO7 supports NF-kappaB-mediated transcription by interacting with the transactivation domain of the p65 subunit. J. Biol. Chem..

[28] Hoesel B, Schmid JA (2013). The complexity of NF-kappaB signaling in inflammation and cancer. Mol. Cancer.

[29] Bronte G (2014). Are erlotinib and gefitinib interchangeable, opposite or complementary for non-small cell lung cancer treatment? Biological, pharmacological and clinical aspects. Crit. Rev. Oncol. Hematol..

[30] Ochi N (2014). Src mediates ERK reactivation in gefitinib resistance in non-small cell lung cancer. Exp. Cell Res..

[31] Scheller J, Chalaris A, Schmidt-Arras D, Rose-John S (2011). The pro- and anti-inflammatory properties of the cytokine interleukin-6. Biochim. Biophys. Acta.

[32] Heinrich PC (2003). Principles of interleukin (IL)-6-type cytokine signalling and its regulation. Biochem. J..

[33] Kyriakis JM, Avruch J (1996). Sounding the alarm: protein kinase cascades activated by stress and inflammation. J. Biol. Chem..

[34] Guan Z, Baier LD, Morrison AR (1997). p38 mitogen-activated protein kinase down-regulates nitric oxide and up-regulates prostaglandin E2 biosynthesis stimulated by interleukin-1beta. J. Biol. Chem..

[35] Meini A, Sticozzi C, Massai L, Palmi M (2008). A nitric oxide/Ca(2+)/calmodulin/ERK1/2 mitogen-activated protein kinase pathway is involved in the mitogenic effect of IL-1beta in human astrocytoma cells. Br. J. Pharmacol..

[36] Morgillo F, Della Corte CM, Fasano M, Ciardiello F (2016). Mechanisms of resistance to EGFR-targeted drugs: lung cancer. ESMO Open.

[37] Sequist LV (2011). Genotypic and histological evolution of lung cancers acquiring resistance to EGFR inhibitors. Sci. Transl. Med.

[38] Ware KE (2013). A mechanism of resistance to gefitinib mediated by cellular reprogramming and the acquisition of an FGF2-FGFR1 autocrine growth loop. Oncogenesis.

[39] Libermann TA, Baltimore D (1990). Activation of interleukin-6 gene expression through the NF-kappa B transcription factor. Mol. Cell. Biol..

[40] Elliott CL, Allport VC, Loudon JA, Wu GD, Bennett PR (2001). Nuclear factor-kappa B is essential for up-regulation of interleukin-8 expression in human amnion and cervical epithelial cells. Mol. Hum. Reprod..

[41] Mihara M, Hashizume M, Yoshida H, Suzuki M, Shiina M (2012). IL-6/IL-6 receptor system and its role in physiological and pathological conditions. Clin. Sci. (Lond.).

[42] Long X (2016). IL-8, a novel messenger to cross-link inflammation and tumor EMT via autocrine and paracrine pathways (Review). Int. J. Oncol..

[43] Kutikov A (2011). Interleukin-6: a potential biomarker of resistance to multitargeted receptor tyrosine kinase inhibitors in castration-resistant prostate cancer. Urology.

[44] Liu YN (2015). IL-8 confers resistance to EGFR inhibitors by inducing stem cell properties in lung cancer. Oncotarget.

[45] Coco S (2015). Afatinib resistance in non-small cell lung cancer involves the PI3K/AKT and MAPK/ERK signalling pathways and epithelial-to-mesenchymal transition. Target Oncol..

[46] Blakely CM (2015). NF-kappaB-activating complex engaged in response to EGFR oncogene inhibition drives tumor cell survival and residual disease in lung cancer. Cell Rep..

