# Supplementary material for: RNF25 promotes gefitinib resistance in EGFR-mutant NSCLC cells by inducing NF-κB-mediated ERK reactivation
Source: Cell Death Dis. 2018 May 22;9(6):587. doi: 10.1038/s41419-018-0651-5 (PMC5964247; doi:10.1038/s41419-018-0651-5)
Supplement: Supplementary file 1 — Supplemental Table 1 [file 41419_2018_651_MOESM1_ESM.docx]

| **Supplemental Table 1.** Characteristics of the two patient-derived NSCLC cells. | | | | | |
| --- | --- | --- | --- | --- | --- |
| Cell name | Origin | Cancer | Genotyping | Treatments | Note |
|  |  |  |  |  |  |
| YL-05 | Pleural effusion | NSCLC, ADC, Stage IV (Metastasis to lung, liver, bone); Never smoker | EGFR (exon19del) | /t TC (2011.1.31~4.11) (PR)→PD 2011.7; /t CS7017+Erlotinib (2011.8.3~2012.2.29) PR→PD;  /t Alimta (2012.3.5~3.26) →PD;  /t BIBW2992 (2012.5.7~7.29) PR→PD;  /t Gemzar (2012.12.3~2013.1.2) →PD on Docetaxel 60 (1/23~) | death (2013.3.28) |
| YL-08 | Pleural effusion | NSCLC, ADC, Stage IV (Metastasis to neck LNs, bone, pericardial); 30PYRS ex-smoker (2011.quit) | Wild type EGFR; ALK positive | /t FP (12.6.20-12.9.15); /t pall RT on T11,12,L3, total 3750Gy/15fx (2012.10.23~11.12); /t Alimta (2012.12.18~2013.3.13) SD→PD; /t Paclitaxel/Carboplatin (13.4.12); CTx refuse. | death (2013.5.24) |
|  | | | | | |
| l  /t : treatment | | | | | |
| l  PR : partial response | | | | | |
| l  PD : progressive diseases | | | | | |
| l  SD : stable diseases | | | | | |
| l  RT : radiation | | | | | |
| l  CS-7017 : a selective peroxisome proliferator-activated receptor gamma agonist of thiazolidinedione class | | | | | |
| l  Alimta : brand name of pemetrexed; a chemotherapy drug manufactured and marketed by Eli Lilly | | | | | |
| l  BIBW2992 : Afatinib; an irreversible EGFR/HER2 inhibitor | | | | | |
| l  Gemzar : brand name of gemcitabine, a chemotherapy medication | | | | | |
| l  Decetaxsol : Taxotere, a chemotherapy medication | | | | | |
| l  CTX : cyclophosphamide, a type of alkylating agent | | | | | |
| l  TC : paclitaxel/carboplatin | | | | | |
| l  FP : 5-FU/DDP | | | | | |
