# Supplementary material for: RNF25 promotes gefitinib resistance in EGFR-mutant NSCLC cells by inducing NF-κB-mediated ERK reactivation
Source: Cell Death Dis. 2018 May 22;9(6):587. doi: 10.1038/s41419-018-0651-5 (PMC5964247; doi:10.1038/s41419-018-0651-5)
Supplement: Supplementary file 3 — Supplemental Figure Legends [file 41419_2018_651_MOESM3_ESM.docx]

**Supplemental Figure Legends**

**Supplemental Figure 1. Validation of selected candidates from the synthetic lethal screen. A.** Reproducibility test of the shRNA library screening. Correlation analysis was carried out using the sequence analysis data for two replicate experiments for each group. **B.** H1650 cells were lentivirally infected with pooled shRNAs for each of the candidate synthetic lethality genes as selected in Figure 1B. Knockdown of the candidate gene expression was confirmed by real-time RT-PCR. Values are mean ± SD of two independent experiments. **C.** Characteristics of EGFR mutant NSCLC cell lines used in this study. The gefitinib sensitivity of each cell line was determined by treating the cells with varying concentrations of gefitinib for 72 h. Values are mean ± SD of three independent experiments.

**Supplemental Figure 2. Determination of RNF25 protein levels in samples.** **A.** RNF25 protein levels were determined in the cells transfected with RNF25-specific siRNAs (related to Figure 2A). **B.** H1650 cells were transduced with shRNA (related to Figure 2B). **C.** H1650 cells were transduced with shRNF25 or RNF25 gene (related to Figure 2C). **D.** HCC827 or PC-9 cells were transfected with RNF25 gene (related to Figures 2D and 3D).

**Supplemental Figure 3. Determination of the time course of EGFR/ERK signal changes in H1650 cells following gefitinib treatment.** H1650 cells were treated with gefitinib (5 μM) for the indicated time period and then subjected to western blot analysis. Values are mean ± SD of three independent experiments.

**Supplemental Figure 4. Activation of NF-κB by gefitinib treatment in H1650 cells.** H1650 cells were treated with gefitinib or vehicle for 24 h, and NF-κB activity was determined in by measuring luciferase activity using a Dual‐Luciferase Reporter Assay System (Promega, Madison, WI, USA). Data were normalized with Renilla luciferase activity. Values are mean ± SD of three independent experiments.

**Supplemental Figure 5. RNF25 depletion sensitizes gefitinib-resistant PDCs to gefitinib. A.** Determination of EGFR/ERK signal changes in gefitinib-resistant lung cancer PDC YL05 cells following gefitinib treatment. YL05 cells were treated with gefitinib (5 μM) for the indicated time period and then subjected to western blot analysis. β-Actin was used as a loading control. **B.** Gefitinib-resistant lung cancer PDC YL08 cells were infected with shRNF25- or pLKO-harboring lentivirus and treated with gefitinib (5 μM) or DMSO for 3 days. Protein levels were analyzed by western blotting. β-Actin was used as a loading control. **C-D.** Gefitinib-resistant lung cancer PDCs were infected with shRNF25- or pLKO-harboring lentivirus and treated with gefitinib (5 μM) or DMSO for 10 days (**C**) or 27 days (**D**). Cell growth was measured by colony formation assay after staining with 0.5% crystal violet (**C**), or by anchorage-independent growth assay in soft agar (**D**). Statistical significance was determined by the Student’s *t* test (**p* < 0.05).
