# Supplementary figures and images for: RNF25 promotes gefitinib resistance in EGFR-mutant NSCLC cells by inducing NF-κB-mediated ERK reactivation
Source: Cell Death Dis. 2018 May 22;9(6):587. doi: 10.1038/s41419-018-0651-5 (PMC5964247; doi:10.1038/s41419-018-0651-5)

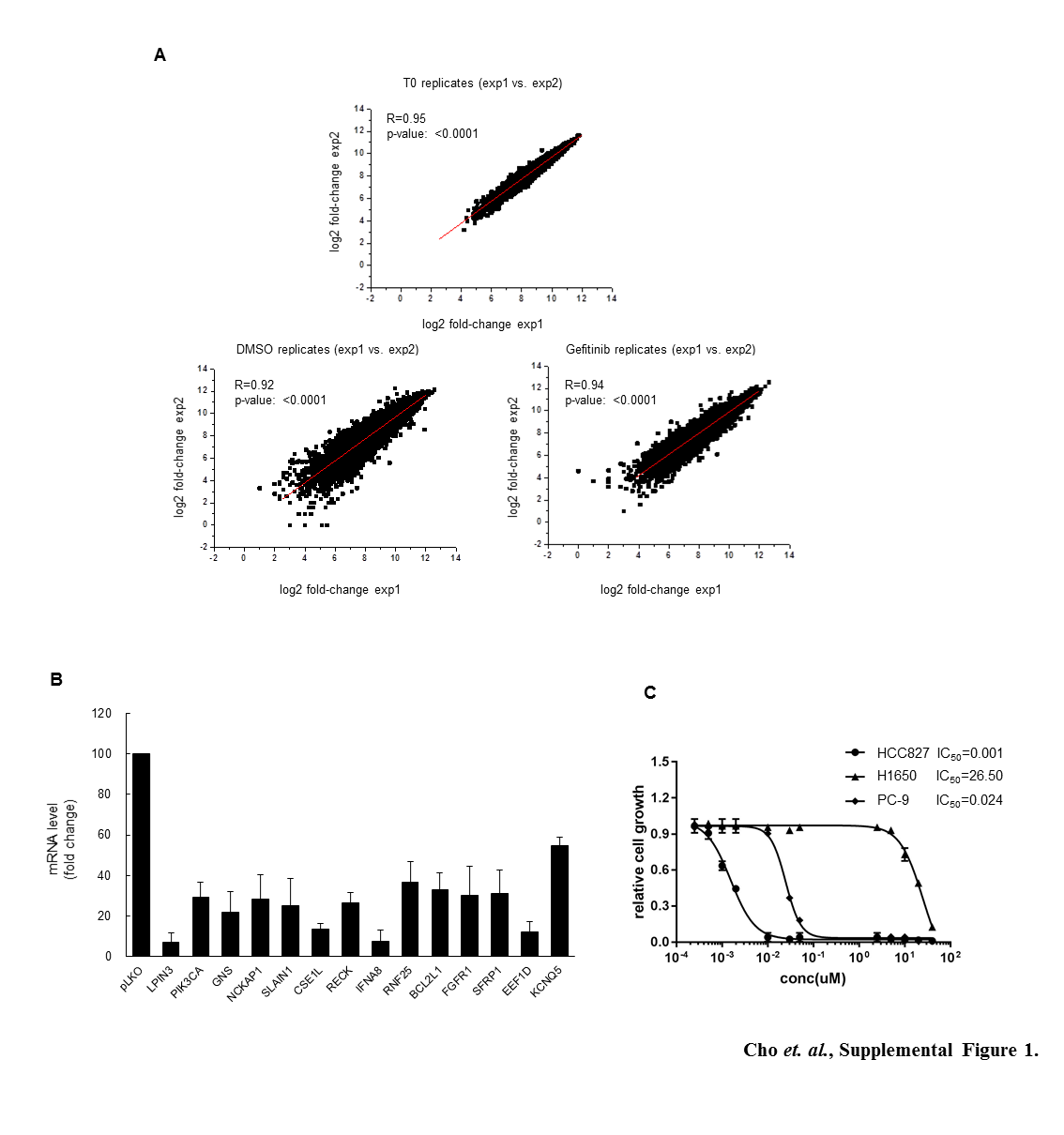

Supplement: Supplementary file 4 — Supplemental Figure 1 [file 41419_2018_651_MOESM4_ESM.tif]

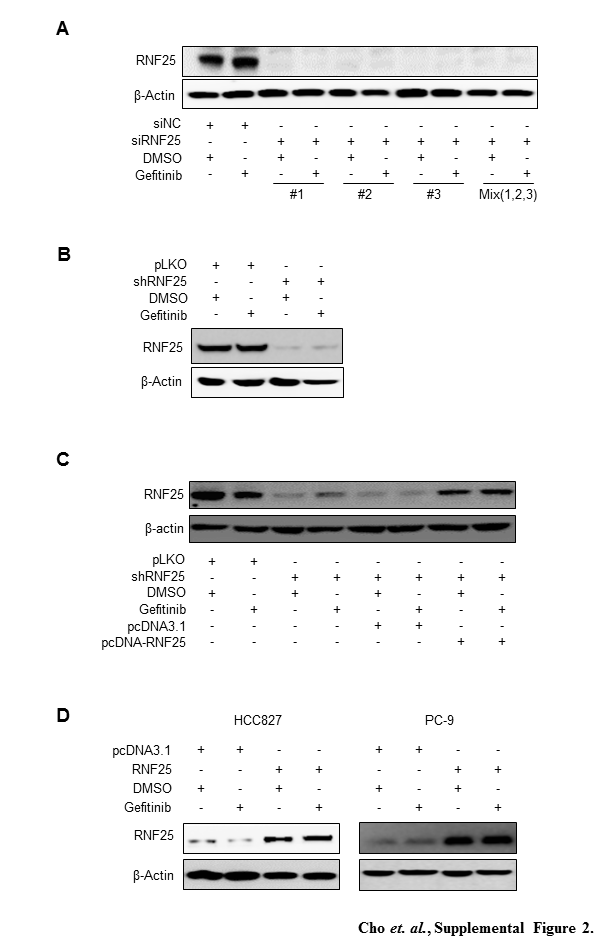

Supplement: Supplementary file 5 — Supplemental Figure 2 [file 41419_2018_651_MOESM5_ESM.tif]

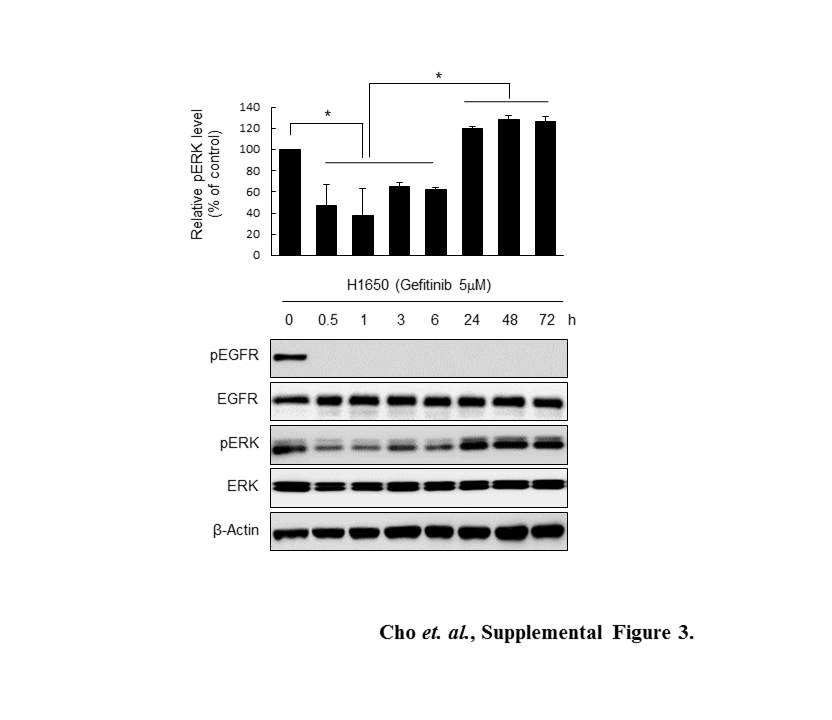

Supplement: Supplementary file 6 — Supplemental Figure 3 [file 41419_2018_651_MOESM6_ESM.tif]

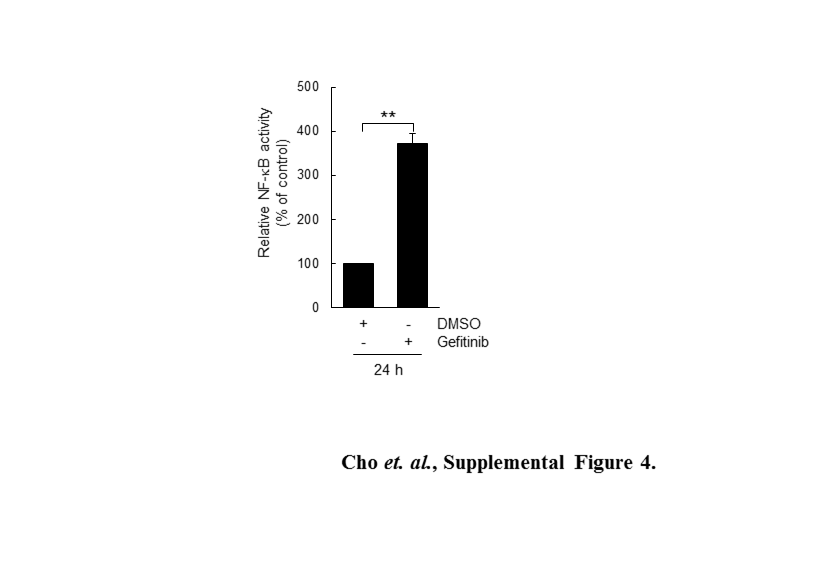

Supplement: Supplementary file 7 — Supplemental Figure 4 [file 41419_2018_651_MOESM7_ESM.tif]

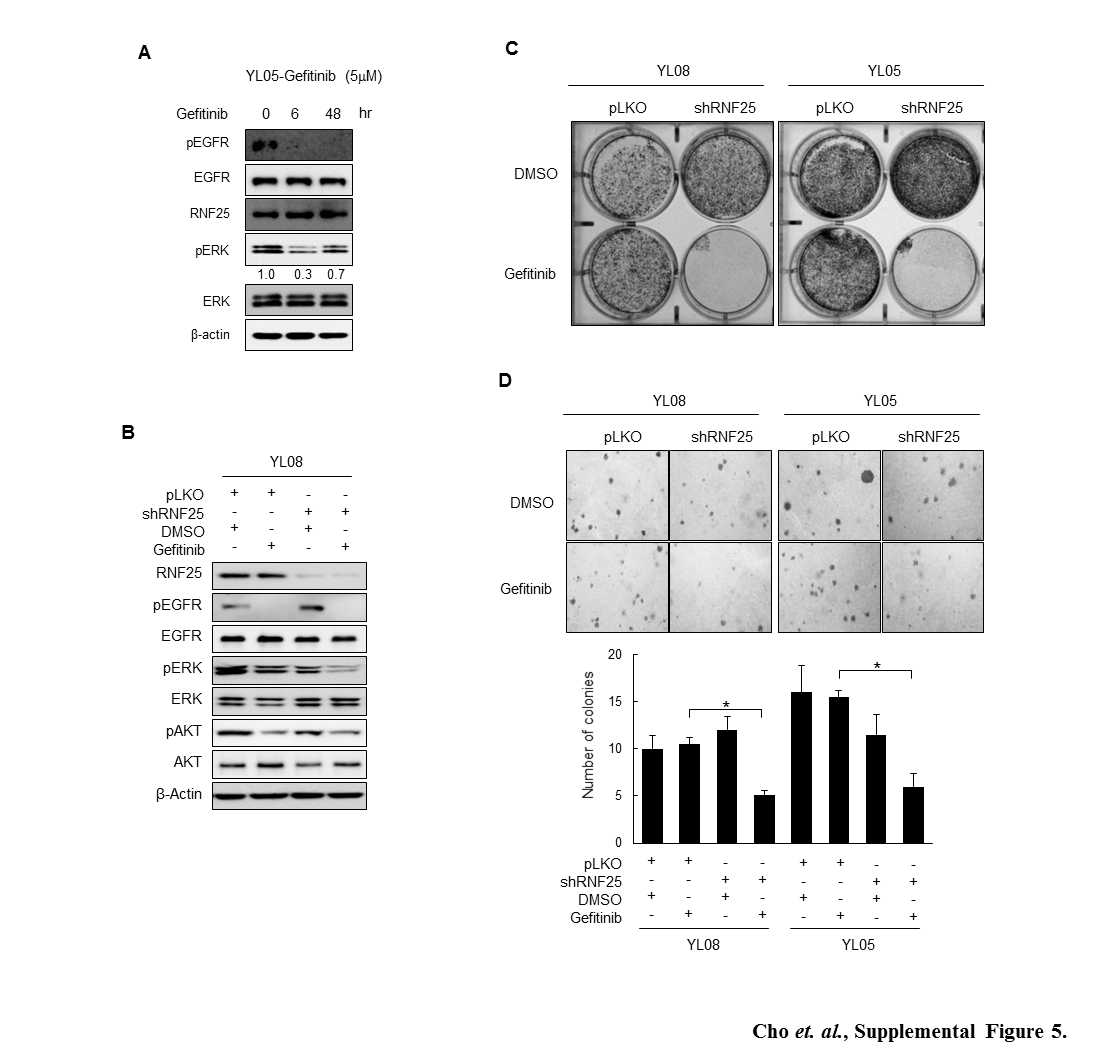

Supplement: Supplementary file 8 — Supplemental Figure 5 [file 41419_2018_651_MOESM8_ESM.tif]
